# Emergence of Good Conduct, Scaling and Zipf Laws in Human Behavioral Sequences in an Online World

**DOI:** 10.1371/journal.pone.0029796

**Published:** 2012-01-12

**Authors:** Stefan Thurner, Michael Szell, Roberta Sinatra

**Affiliations:** 1 Section for Science of Complex Systems, Medical University of Vienna, Vienna, Austria; 2 Santa Fe Institute, Santa Fe, New Mexico, United States of America; 3 IIASA, Laxenburg, Austria; 4 Dipartimento di Fisica e Astronomia, Università di Catania and INFN, Catania, Italy; 5 Laboratory for Complex Systems, Scuola Superiore di Catania, Catania, Italy; University of Zaragoza, Spain

## Abstract

We study behavioral action sequences of players in a massive multiplayer online game. In their virtual life players use eight basic actions which allow them to interact with each other. These actions are communication, trade, establishing or breaking friendships and enmities, attack, and punishment. We measure the probabilities for these actions conditional on previous taken and received actions and find a dramatic increase of negative behavior immediately after receiving negative actions. Similarly, positive behavior is intensified by receiving positive actions. We observe a tendency towards anti-persistence in communication sequences. Classifying actions as positive (good) and negative (bad) allows us to define binary ‘world lines’ of lives of individuals. Positive and negative actions are persistent and occur in clusters, indicated by large scaling exponents 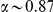 of the mean square displacement of the world lines. For all eight action types we find strong signs for high levels of repetitiveness, especially for negative actions. We partition behavioral sequences into segments of length 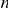 (behavioral ‘words’ and ‘motifs’) and study their statistical properties. We find two approximate power laws in the word ranking distribution, one with an exponent of 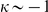 for the ranks up to 100, and another with a lower exponent for higher ranks. The Shannon 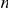-tuple redundancy yields large values and increases in terms of word length, further underscoring the non-trivial statistical properties of behavioral sequences. On the collective, societal level the timeseries of particular actions per day can be understood by a simple mean-reverting log-normal model.

## Introduction

Societies can be seen as individuals interacting through a *multiplex network* (MPN), i.e. a superposition of several social networks defined on the same set of nodes (individuals) [1], [2]. Different types of networks correspond to different types of social interactions. For example the communication sub-network of the MPN is the network whose links correspond to the exchange of information by means of emails, telephone calls, or letters. Another subnetwork is the trading network, where goods or services are exchanged between individuals, in exchange for other goods, money, or –rarely– for nothing. Each of these interactions usually needs an initial action taken by one of the subjects involved in the exchange, the *sender*, and a target to receive it, the *recipient*. Actions can (but do not have to) be reciprocated, so that in general the MPN consists of a set of directed and weighted subnetworks. The MPN is a highly non-trivial dynamical object. The different social networks within the MPN are not independent but strongly influence each other through a *network-network interaction*. To understand systemic properties of societies it is essential to detect and quantify the organizational principles behind such mutual influences. The MPN is an example of a *co-evolving* structure: on one hand the actions of individuals shape and define the topological structure of the MPN. On the other hand the topology of the MPN constrains and influences the possible actions which take place on the MPN. In general the MPN of a society can not be observed due to immense requirements on synchronized data acquisition. Despite these difficulties, the analysis of *small-scale* MPNs has a tradition in the social sciences [1], [3]–[5]. Concerning large-scale studies, recently there have been significant achievements in understanding a number of massive social networks on a quantitative basis, such as the cell phone communication network [6]–[8], features of the world-trade network [9], [10], email networks [11], the network of financial debt [12] and the network of financial flows [13]. The integration of various dynamical networks of an entire society has so-far been beyond the scope of any realistic data source. However with the increasing availability of vast amounts of electronic fingerprints people leave throughout their lifes, this situation is about to change. Online sources are capturing more and more aspects of life, boosting our understanding of collective human behavior [14], [15]. One particular source where *complete* behavioral multiplex data is available on the society level are massive multiplayer online games (MMOGs). In MMOGs hundreds of thousands of players meet online in a ‘virtual life’ where their actions can be easily studied [16]. Players have to gain their living through economic activity and usually are integrated in several types of social networks. In such games communication networks, friendship and enmity networks have been studied, initially as separated entities [17], [18]. In [2] trading, aggression and punishment networks have been added to the analysis and first measurements on mutual network-network influences were reported.

In this paper we do not focus on the full MPN but on the dynamics (actions) taking place on its nodes. We report on the nature of sequences of human behavioral actions in a virtual universe of a MMOG. There sequential behavioral data is available on the scale of an entire society, which is in general impossible to obtain. The unique data of the online game Pardus [19] allows to unambiguously track all actions of all players over long time periods. We focus on the stream of eight types of actions which are translated into an 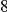-letter alphabet. This *code* of actions of individual players is then analyzed by means of standard timeseries approaches as have been used, for example, in DNA sequence analyzes [20]–[22].

## Materials and Methods

### The game

The dataset contains practically all actions of all players of the MMOG Pardus since the game went online in 2004 [19]. Pardus is an open-ended online game with a worldwide player base of currently more than 370,000 people. Players live in a virtual, futuristic universe in which they interact with others in a multitude of ways to achieve their self-posed goals [23]. Most players engage in various economic activities typically with the (self-posed) goal to accumulate wealth and status. Social and economical decisions of players are often strongly influenced and driven by social factors such as friendship, cooperation, and conflict. Conflictual relations may result in aggressive acts such as attacks, fights, punishment, or even destruction of another player's means of production or transportation. The dataset contains longitudinal and relational data allowing for a complete and dynamical mapping of multiplex relations of the entire virtual society, over 1238 days. The behavioral data are free of ‘interviewer-bias’ or laboratory effects since users are not reminded of their actions being logged during playing. The longitudinal aspect of the data allows for the analysis of dynamical aspects such as the emergence and evolution of network structures. It is possible to extract multiple social relationships between a fixed set of humans [2].

The game Pardus [19] is sectioned into three independent ‘universes’. Here we focus on the ‘Artemis’ universe, in which we recorded player actions over the first 1,238 consecutive days of the universe's existence. Communication between any two players can take place directly, by using a one-to-one, e-mail-like private messaging system, or indirectly, by meeting in built-in chat channels or online forums. For the player action sequences analyzed we focus on one-to-one interactions between players only, and discard indirect interactions such as e.g. participation in chats or forums [24]. Players can express their sympathy (distrust) toward other players by establishing so-called friendship (enmity) links. These links are only seen by the player marking another as a friend (enemy) and the respective recipient of that link. For more details on the game, see [18], [19]. From all sequences of all 34,055 Artemis players who performed or received an action at least once within 1,238 days, we removed players with a life history of less than 1000 actions, leading to the set of the most active 1,758 players which are considered throughout this work. All data used in this study is fully anonymized; the authors have the written consent to publish from the legal department of the Medical University of Vienna.

### Human behavioral sequences

**Figure 1:**
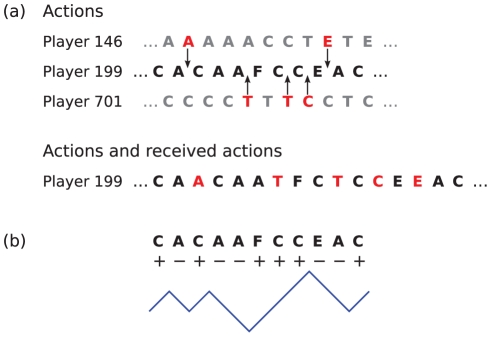
Short segment of action sequences of three players, 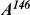, 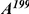, and 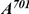. (a). Some actions of players 146 and 701 are directed toward player 199. This results in a sequence of received-actions for player 199, 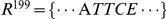. The combined sequence of actions (originated from - and directed to) player 199, 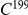, is shown in the last line; red letters mark actions from others directed to player 199. (b) Schematic illustration showing the definition of a binary walk in ‘good-bad’ action space (good-bad ‘world line’). A positive action (C, T, F or X) means an upward move, a negative action (A, B, D and E) is a downward move. Good people have rising world-lines.

We consider eight different actions every player can execute at any time. These are communication (C), trade (T), setting a friendship link (F), removing an enemy link (forgiving) (X), attack (A), placing a bounty on another player (punishment) (B), removing a friendship link (D), and setting an enemy link (E). While C, T, F and X can be associated with *positive* (good) actions, A, B, D and E are hostile or *negative* (bad) actions. We classify communication as positive because only a negligible part of communication takes place between enemies [18]. Segments of action sequences of three players (146, 199 and 701) are shown in the first three lines of Fig. 1 (a).

We consider three types of sequences for any particular player. The first is the stream of 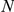 consecutive actions 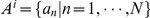 which player 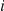 performs during his ‘life’ in the game. The second sequence is the (time-ordered) stream of actions that player 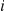 receives from all the other players in the game, i.e. all the actions which are directed towards player 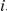 We denote by 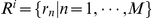 received-action sequences. Finally, the third sequence is the time-ordered combination of player 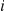
's actions and received-actions, which is a chronological sequence from the elements of 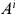 and 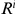 in the order of occurrence. The combined sequence we denote by 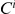; its length is 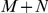, see also Fig. 1 (a). The 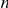
th element of one of these series is denoted by 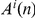, 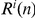, or 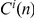. We do not consider the actual time between two consecutive actions which can range from milliseconds to weeks, rather we work in ‘action-time’.

If we assign 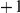 to any positive action C, T, F or X, and 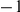 to the negative actions A, B, D and E, we can translate a sequence 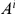 into a symbolic binary sequence 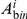. From the cumulative sum of this sequence a ‘world line’ or ‘random walk’ for player 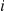 can be generated, 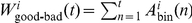, see Fig. 1 (b). Similarly, we define a binary sequence from the combined sequence 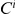, where we assign 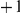 to an executed action and 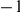 to a received-action. This sequence we call 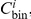 its cumulative sum, 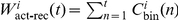 is the ‘action-receive’ random-walk or world line. Finally, we denote the number of actions which occurred during a day in the game by 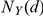, where 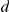 indicates the day and 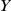 stands for one of the eight actions.

## Results

**Figure 2:**
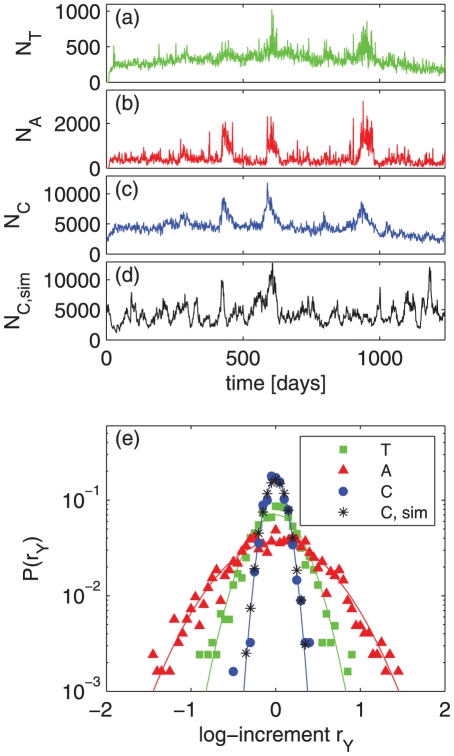
Timeseries of the daily number of (a) trades, (b) attacks, (c) communications in the first 1238 days in the game. Clearly a mean reverting tendency of three processes can be seen. (d) Simulation of a model timeseries, Eq. (1), with 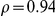. We use the values from the 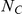 timeseries, 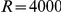, and standard deviation 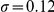. Compare with the actual 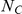 in (c). The free parameter in the model is 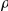. Parameters are from Tab. 1. Mean reversion and log-normality motivate the model presented in Eq. (1). (e) The distributions of log-increments 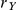 of the processes and the model. All follow approximate Gaussian distribution functions.

The number of occurrences of the various actions of all players over the entire time period is summarized in Tab. 1 (first line). Communication is the most dominant action, followed by attacks and trading which are each about an order of magnitude less frequent. The daily number of all communications, trades and attacks, 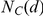, 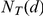 and 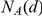 is shown in Fig. 2 (a), (b) and (c), respectively. These processes are reverting around a mean, 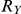. All processes of actions show an approximate Gaussian statistic of its log-increments, 
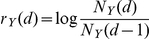
. The first 4 moments of the 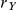 series are listed in Tab. 1. The relatively large kurtosis for 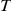 and 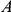 results from a few extreme outliers. The distribution of log-increments for the 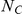, 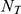 and 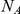 timeseries are shown in Fig. 2 (d). The lines are Gaussians for the respective mean and standard deviation from Tab. 1. As maybe the simplest mean-reverting model with approximate log-normal distributions, we propose
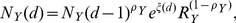
(1)where 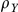 is the mean reversion coefficient, 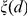 is a realization of a zero mean Gaussian random number with standard deviation 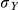, and 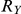 is the value to which the process 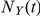 reverts to. 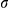 is given by the third line in Tab. 1. Note that this is an AR(1) process (Ornstein-Uhlenbeck process in discrete time) in logarithmic variables.

**Table 1:** First row: total number of actions by all players (with at least 1000 actions) in the Artemis universe of the Pardus game.

	D	B	A	E	F	C	T	X
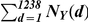	26,471	9,914	558,905	64,444	82,941	5,607,060	393,250	20,165
mean	0.002	0.001	0.004	−0.002	−0.002	0.000	0.003	0.002
std	1.13	0.79	0.54	0.64	0.35	0.12	0.28	0.94
skew	0.12	0.26	0.35	0.08	0.23	0.11	1.00	−0.01
kurtosis	3.35	3.84	6.23	3.67	3.41	3.76	13.89	3.72

Further rows: first 4 moments of 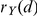, the distribution of the log-increments of the 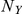 processes (see text). Approximate log-normality is indicated. The large values of kurtosis for 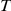 and 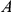 result from a few extreme outliers.

### Transition probabilities

**Figure 3:**
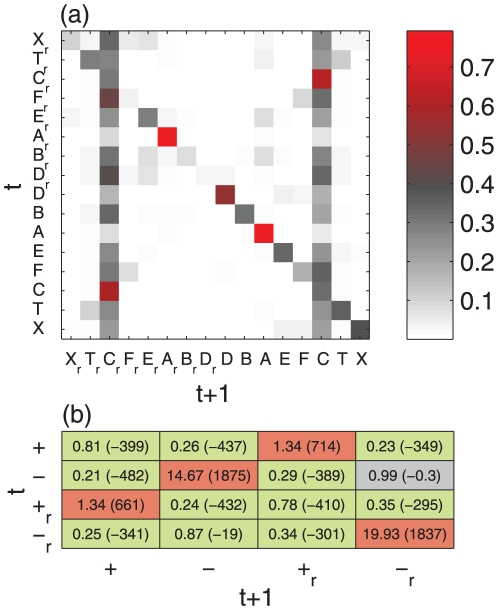
Transition probabilities 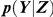 for actions (and received-actions) 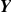 at a time 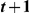, given that a specific action 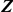 was executed or received in the previous time-step 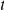. (a). Received-actions are indicated by a subscript 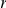. Normalization is such that rows add up to one. The large values in the diagonal signal that human actions are highly clustered or repetitive. Large values for 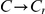 and 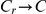 reveal that communication is a tendentially anti-persistent activity – it is more likely to receive a message after one sent a message and vice versa, than to send or to receive two consecutive messages. (b) The ratio 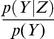, shows the influence of an action 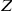 at a previous time-step 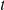 on a following action 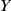 at a time 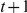, where 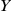 and 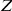 can be positive or negative actions, executed or received (received actions are indicated by the subscript 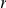). In brackets, we report the *Z*-score (significance in number of standard deviations) in respect to a sample of 100 randomized versions of the dataset. The cases for which the transition probability is significantly higher (lower) than expected in uncorrelated sequences are highlighted in red (green). Receiving a positive action after performing a positive action is highly over-represented, and vice versa. Performing (receiving) a negative action after performing (receiving) another negative one is also highly over-represented. Performing a negative action has no influence on receiving a negative action next. All other combinations are strongly under-represented, for example after performing a negative action it is very unlikely to perform a positive action with respect to the uncorrelated case.

With 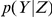 we denote the probability that an action of type 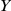 follows an action of type 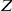 in the behavioral sequence of a player. 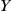 and 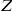 stand for any of the eight actions, executed or received (received is indicated by a subscript 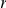). In Fig. 3 (a) the transition probability matrix 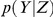 is shown. The 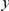 axis of the matrix indicates the action (or received-action) happening at a time 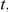 the probabilities for the actions (or received-actions) that immediately follow are given in the corresponding horizontal place.

This transition matrix specifies to which extent an action or a received action of a player is influenced by the action that was done or received at the previous time-step. In fact, if the behavioral sequences of players had no correlations, i.e. the probability of an action, received or executed, is independent of the history of the player's actions, the transition probability 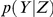 simply is 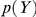, i.e. to the probability that an action or received action 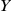 occurs in the sequence is determined by its relative frequency only. Therefore, deviations of the ratio 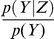 from 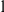 indicate correlations in sequences. In Fig. 3 (b) we report the values of 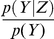 for actions and received actions (received actions are indicated with the subscript 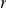) classified only according to their positive (+) or negative (−) connotation. In brackets we report the *Z*-score with respect to the uncorrelated case. We find that the probability to perform a good action is significantly higher if at the previous time-step a positive action has been received. Similarly, it is more likely that a player is the target of a positive action if at the previous time-step he executed a positive action. Conversely, it is highly unlikely that after a good action, executed or received, a player acts negatively or is the target of a negative action. Instead, in the case a player acts negatively, it is most likely that he will perform another negative action at the following time-step, while it is highly improbable that the following action, executed or received, will be positive. Finally, in the case a negative action is received, it is likely that another negative action will be received at the following time-step, while all other possible actions and received actions are under-represented. The high statistical significance of the cases 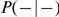 and 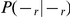 hints toward a high persistence of negative actions in the players' behavior, see below.

Another finding is obtained by considering only pairs of received actions followed by performed actions. This approach allows to quantify the influence of received actions on the performed actions of players. For these pairs we measure a probability of 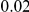 of performing a negative action after a received positive action. This value is significantly lower compared to the probability of 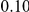 obtained for randomly reshuffled sequences. Similarly, we measure a probability of 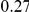 of performing a negative action after a received negative action. Note that this result is not in contrast with the values in Fig. 3 (b), since only pairs made up of received actions and performed actions are taken into account. Our results agree with a recent study where the emotional content of posts in online forums was analyzed similarly [24].

### World lines

**Figure 4:**
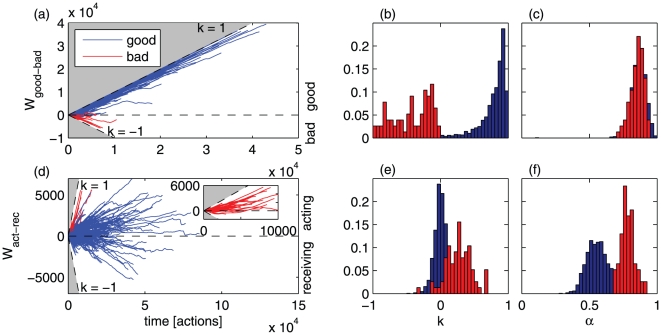
World lines of good-bad action random walks of the 1,758 most active players (a), distribution of their slopes 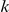 (b), and of their scaling exponents 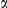 (c). By definition, players who perform more good (bad) than bad (good) actions have the endpoints of their world lines above (below) 0 in (a) and only fall into the 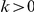 (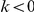) category in (b). (d) World lines of action-received random walks, (e) distribution of their slopes 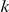 and (f) of their scaling exponents 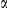. The inset in (d) shows only the world lines of bad players. These players are typically dominant, i.e. they perform significantly more actions than they receive. In total the players perform many more good than bad actions and are strongly persistent with good as well as with bad behavior, see (c), i.e. actions of the same type are likely to be repeated.

The world lines 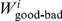 of good-bad action sequences are shown in Fig. 4 (a), the action-reaction world lines in Fig. 4 (b). As a simple measure to characterize these world lines we define the slope 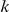 of the line connecting the origin of the world line to its end point (last action of the player). A slope of 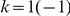 in the good-bad world lines 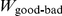 indicates that the player performed only positive (negative) actions. The slope 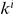 is an approximate measure of ‘altruism’ for player 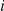. The histogram of the slopes for all players is shown in Fig. 4 (b), separated into good (blue) and bad (red) players, i.e. players who have performed more good than bad actions and vice versa. The mean and standard deviation of slopes of good, bad, and all players are 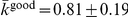, 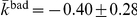, and 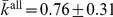, respectively. Simulated random walks with the same probability 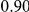 of performing a positive action yield a much lower variation, 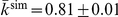, pointing at an inherent heterogeneity of human behavior. For the combined action–received-action world line 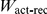 the slope is a measure of how well a person is integrated in her social environment. If 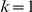 the person only acts and receives no input, she is ‘isolated’ but dominant. If the slope is 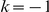 the person is driven by the actions of others and does never act nor react. The histogram of slopes for all players is shown in Fig. 4 (e). Most players are well within the 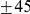 degree cone. Mean and standard deviation of slopes of good, bad, and all players are 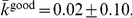 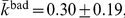 and 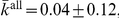 respectively. Bad players are tendentially dominant, i.e. they perform significantly more actions than they receive. Simulated random walks with equal probabilities for up and down moves for a sample of the same sequence lengths, we find again a much narrower distribution with slope 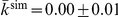.

As a second measure we use the mean square displacement of world lines to quantify the persistence of action sequences,
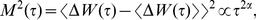
(2)where 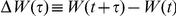 and 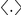 is the average over all 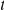. The asymptotic behavior of 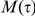 yields information about the ‘persistence’ of a world line. 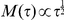 is the pure diffusion case, 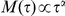 with scaling exponent 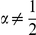 indicates persistence for 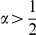, and anti-persistence for 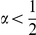. Persistence means that the probability of making an up(down) move at time 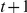 is larger(less) than 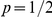, if the move at time 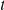 was an up move. For calculating the exponents 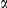 we use a fit range of 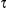 between 5 and 100. We checked from the mean square displacement of single world lines that this fit range is indeed reasonable.

The histogram of exponents 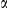 for the good-bad random walk, separated into good (blue) and bad (red) players, is shown in Fig. 4 (c), for the action–received-action world line in (f). In the first case strongly persistent behavior is obvious, in the second there is a slight tendency towards persistence. Mean and standard deviation for the good-bad world lines are 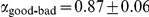, for the action-received actions 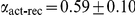. Simulated sequences of random walks have – as expected by definition – an exponent of 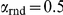, again with a very small standard deviation of about 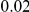. Figure 4 (a) also indicates that the lifetime of players who use negative actions frequently is short. The average lifetime for players with a slope 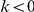 is 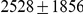 actions, compared to players with a slope 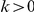 with 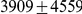 actions. The average lifetime of the whole sample of players is 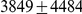 actions.

### Motifs, Entropy and Zipf law

By considering all the sequences of actions 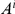 of all possible players 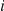, we have an ensemble which allows to perform a motif analysis [25]. We define a 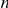-string as a subsequence of 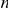 contiguous actions. An 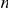-motif is an 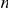-string which appears in the sequences with a probability higher than expected, after lower-order correlations have been properly removed.

Across the entire ensemble, 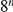 different 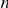-strings can appear, each of them occurring with a different probability. The frequency, or observed probability, of each 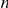-string can be compared to its expected probability of occurrence, which can be estimated on the basis of the observed probability of lower order strings, i.e. on the frequency of 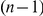-strings. For example, the expected probability of occurrence of a 2-string 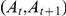 is estimated as the product of the observed probability of the single actions 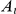 and 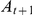, 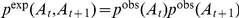. Similarly, the probability of a 3-string 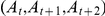 to occur can be estimated as 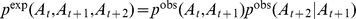, where 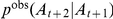 is the conditional probability to have action 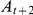 following action 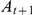. By definition of conditional probability, one has 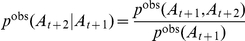 (see [25] for details). A 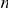-motif in the ensemble is then defined as a 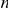-string whose observed probability of occurrence is significantly higher than its expected probability.

We computed the observed and expected probabilities 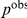 and 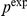 for all 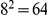 2-strings and for all 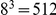 3-strings, focusing on those 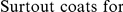-strings with the highest ratio 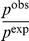. Higher orders are statistically not feasible due to combinatorial explosion. We find that the 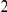-motifs in the sequences of actions 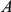 are clusters of same letters: BB, DD, XX, EE, FF, AA with ratios 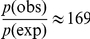, 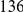, 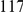, 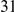, 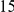, 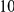, respectively. This observation is consistent with the previous first-order observation that actions cluster. The most significant 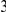-motifs however are (with two exceptions) palindromes: EAX, DAF, DCD, DAD, BGB, BFB, with ratios 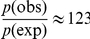, 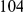, 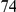, 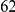, 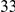, 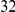, respectively. The exceptions disappear when one considers actions executed on the same screen in the game as equivalent, i.e. setting or removing friends or enemies: F, D, E, X. This observation hints towards processes where single actions of one type tend to disrupt a flow of actions of another type.

**Figure 5:**
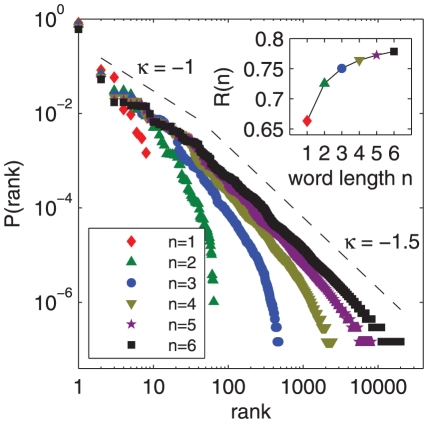
Rank ordered probability distribution of 1 to 6 letter words. Slopes of 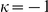 and 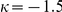 are indicated for reference. The inset shows the Shannon 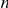-tuple redundancy as a function of word length 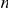.

Finally, we partition the action sequences into 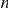-strings (‘words’). Fig. 5 shows the rank distribution of word occurrences of different lengths 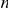. The distribution shows an approximate Zipf law [26] (slope of 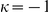) for ranks below 100. For ranks between 100 and 25,000 the scaling exponent approaches a smaller value of about 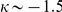. The Shannon 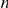-tuple redundancy (see e.g. [20]–[22]) for symbol sequences composed of 8 symbols (our action types) is defined as
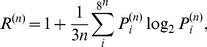
(3)where 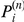 is the probability of finding a specific 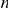-letter word. Uncorrelated sequences yield an equi-distribution, 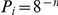, i.e. 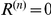. In the other extreme of only one letter being used, 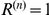. In Fig. 5 (inset) 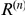 is shown as a function of sequence length 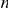. Shannon redundancy is not a constant but increases with 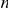 This indicates that Boltzmann-Gibbs entropy might not be an extensive quantity for action sequences [27].

## Discussion

The analysis of human behavioral sequences as recorded in a massive multiplayer online game shows that communication is by far the most dominant activity followed by aggression and trade. Communication events are about an order of magnitude more frequent than attacks and trading events, showing the importance of information exchange between humans. It is possible to understand the collective timeseries of human actions of a particular type (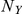) with a simple mean-reverting log-normal model. On the individual level we are able to identify organizational patterns of the emergence of good overall behavior. Transition rates of actions of individuals show that positive actions strongly induces positive *reactions*. Negative behavior on the other hand has a high tendency of being repeated instead of being reciprocated, showing the ‘propulsive’ nature of negative actions. However, if we consider only reactions to negative actions, we find that negative reactions are highly over-represented. The probability of acting out negative actions is about 10 times higher if a person received a negative action at the previous timestep than if she received a positive action. The action of communication is found to be of highly reciprocal ‘back-and-forth’ nature. The analysis of binary timeseries of players (good-bad) shows that the behavior of almost all players is ‘good’ almost all the time. Negative actions are balanced to a large extent by good ones. Players with a high fraction of negative actions tend to have a significantly shorter life. This may be due to two reasons: First because they are hunted down by others and give up playing, second because they are unable to maintain a social life and quit the game because of loneliness or frustration. We interpret these findings as empirical evidence for self organization towards reciprocal, good conduct within a human society. Note that the game allows bad behavior in the same way as good behavior but the extent of punishment of bad behavior is freely decided by the players.

Behavior is highly persistent in terms of good and bad, as seen in the scaling exponent (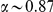) of the mean square displacement of the good-bad world lines. This high persistence means that good and bad actions are carried out in clusters. Similarly high levels of persistence were found in a recent study of human behavior [28]. A smaller exponent (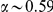) is found for the action–received-action timeseries.

Finally we split behavioral sequences of individuals into subsequences (of length 1–6) and interpret these as behavioral ‘words’. In the ranking distribution of these words we find a Zipf law to about ranks of 100. For less frequent words the exponent in the rank distribution approaches a somewhat smaller exponent of about 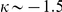. From word occurrence probabilities we further compute the Shannon 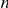-tuple redundancy which yields relatively large values when compared for example to those of DNA sequences [20]–[22]. This reflects the dominance of communication over all the other actions. The 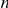-tuple redundancy is clearly not a constant, reflecting again the non-trivial statistical structure of behavioral sequences.
